# Complement Factor H Loss in RPE Cells Causes Retinal Degeneration in a Human RPE-Porcine Retinal Explant Co-Culture Model

**DOI:** 10.3390/biom11111621

**Published:** 2021-11-03

**Authors:** Angela Armento, Aparna Murali, Julia Marzi, Ana C Almansa-Garcia, Blanca Arango-Gonzalez, Ellen Kilger, Simon J Clark, Katja Schenke-Layland, Charmaine A Ramlogan-Steel, Jason C Steel, Marius Ueffing

**Affiliations:** 1 Institute for Ophthalmic Research, Department for Ophthalmology, Eberhard Karls University of Tübingen, 72076 Tübingen, Germany; aparnamuralib@gmail.com (A.M.); ana-cristina.almansa-garcia@uni-tuebingen.de (A.C.A.-G.); blanca.arango-gonzalez@klinikum.uni-tuebingen.de (B.A.-G.); ellen.kilger@uni-tuebingen.de (E.K.); simon.clark@uni-tuebingen.de (S.J.C.); 2 Faculty of Medicine, University of Queensland, Herston, QLD 4006, Australia; c.ramlogan-steel@cqu.edu.au (C.A.R.-S.); j.steel2@uq.edu.au (J.C.S.); 3 Institute of Biomedical Engineering, Department for Medical Technologies and Regenerative Medicine, Eberhard Karls University Tübingen, 72076 Tübingen, Germany; julia.marzi@uni-tuebingen.de (J.M.); katja.schenke-layland@uni-tuebingen.de (K.S.-L.); 4 NMI Natural and Medical Sciences Institute at the University of Tübingen, 72770 Reutlingen, Germany; 5 Cluster of Excellence iFIT (EXC 2180) “Image-Guided and Functionally Instructed Tumor Therapies”, Eberhard Karls University Tübingen, 72076 Tübingen, Germany; 6 Lydia Becker Institute of Immunology and Inflammation, Faculty of Biology, Medicine and Health, University of Manchester, Manchester M13 9PL, UK; 7 Department of Medicine/Cardiology, Cardiovascular Research Laboratories, David Geffen School of Medicine at University of California, Los Angeles, CA 90095, USA; 8 School of Health, Medical and Applied Sciences, Central Queensland University, Brisbane, QLD 4000, Australia

**Keywords:** age-related macular degeneration (AMD), complement factor H (CFH), retinal pigment epithelium (RPE) cells, retinal degeneration, co-culture, porcine retinal explants, Raman, mitochondria, oxidized lipids

## Abstract

Age-related Macular degeneration (AMD) is a degenerative disease of the macula affecting the elderly population. Treatment options are limited, partly due to the lack of understanding of AMD pathology and the lack of suitable research models that replicate the complexity of the human macula and the intricate interplay of the genetic, aging and lifestyle risk factors contributing to AMD. One of the main genetic risks associated with AMD is located on the Complement Factor H (*CFH*) gene, leading to an amino acid substitution in the Factor H (FH) protein (Y402H). However, the mechanism of how this FH variant promotes the onset of AMD remains unclear. Previously, we have shown that FH deprivation in RPE cells, via *CFH* silencing, leads to increased inflammation, metabolic impairment and vulnerability toward oxidative stress. In this study, we established a novel co-culture model comprising *CFH* silenced RPE cells and porcine retinal explants derived from the visual streak of porcine eyes, which closely resemble the human macula. We show that retinae exposed to FH-deprived RPE cells show signs of retinal degeneration, with rod cells being the first cells to undergo degeneration. Moreover, via Raman analyses, we observed changes involving the mitochondria and lipid composition of the co-cultured retinae upon FH loss. Interestingly, the detrimental effects of FH loss in RPE cells on the neuroretina were independent of glial cell activation and external complement sources. Moreover, we show that the co-culture model is also suitable for human retinal explants, and we observed a similar trend when RPE cells deprived of FH were co-cultured with human retinal explants from a single donor eye. Our findings highlight the importance of RPE-derived FH for retinal homeostasis and provide a valuable model for AMD research.

## 1. Introduction

Age-related macular degeneration (AMD) is a complex multifactorial disease that severely compromises visual acuity and eventually leads to irreversible blindness if left untreated [1,2]. It is the leading cause of blindness among the elderly population globally, and the numbers are expected to increase due to the increasing aged population [3]. The region of the eye affected in AMD is the macula, the central region of the retina with the highest number of photoreceptors (PR) that are located on top of RPE cells that themselves sit on top of a thin membrane called Bruch’s membrane (BM), which separates the neuroretina from the choriocapillaris [4]. Up to now, no satisfactory treatment has been discovered for the more common late-stage form of AMD, which is characterized by RPE geographic atrophy (GA). The reasons for that can be associated with the complexity of AMD pathology, which involves a variety of risk factors (aging, genetic predisposition, lifestyle) [5] and the complexity of a multi-layer/multi-cellular structure like the retina. Moreover, the research models used so far do not fully replicate many important aspects of the macula [6]. For example, mono-cellular in vitro studies do not consider the interactions between cell types, and animal models, though largely used for retinal studies, do not replicate the specific properties of the macula, which is unique to humans [7]. A lack of macula-like research models makes it very difficult to investigate the order of events in AMD pathology. Moreover, it is unclear whether rod or cone PR die first and, most importantly, the mechanism behind the cell death of either one of them. Studies from Curcio et al. proposed that rod cell death may precede cone cell death in AMD [8], since in the para-foveal region during the early stages of AMD, the rod PR undergo extensive apoptosis compared to the cones.

Although AMD is triggered by multiple genetic and environmental factors, a large portion of the genetic risk falls into genes of the alternative pathway of the complement system. In particular, one of the most strongly associated genetic variants with AMD risk (rs1061170) resides in the *CFH* gene, which encodes the factor H and factor H-like (FHL-1) proteins: potent regulators of complement activation [9,10]. This genetic variant leads to a coding change in the FH/FHL-1 proteins where a tyrosine residue is replaced by a histidine residue at position 402 (residue 384 in the mature protein [11] and is referred to as the Y402H polymorphism. Furthermore, secreted/extracellular FH/FHL-1 has been described to possess additional functions, and the Y402H polymorphism has been related to different aspects of AMD pathology [4]. For instance, RPE cells are constantly exposed to oxidative stress since the retina consists of a highly oxidized environment, and RPE cells are responsible for the degradation of oxidized phospholipids shed by PR [12]. In this context, FH plays an anti-oxidant protective role [13] directly binding oxidized lipids [14]. However, the 402H high-risk variant of FH protein loses this protective function, probably due to the decreased affinity for oxidized lipids [14], resulting in a reduced ability of the RPE cells to neutralize oxidized lipids, which then accumulate and contribute to drusen formation and therefore promote oxidative stress and inflammation [15,16]. Moreover, RPE cells exist in a complex co-dependent metabolic relationship with PR since the PR rely on RPE cells for the supply of nutrients, and a bioenergetic crisis of RPE cells has been described as part of AMD pathology [17].

Recently, a novel function for intracellular FH has been described in several cell types and place intracellular FH as an important regulator of cell homeostasis [18,19]. iPSC-RPE cells, carrying the 402H high-risk variant of FH, show enlarged mitochondria [20] and reduced mitochondria activity [21], but whether the effects depends on intracellular FH or extracellular FH was not investigated. Our previous studies show that the loss of endogenous/intracellular FH in RPE cells leads to a phenotype very similar to iPSC-RPE cells carrying the 402H high-risk variant of FH. In detail, hTERT-RPE1 cells deprived of endogenous FH via *CFH* silencing are more vulnerable to oxidative stress, show reduced metabolic capacity [18] and modify the microenvironment toward an inflammatory state [22] all predisposing features for AMD.

This study investigates the impact of the endogenous loss of FH in RPE cells on the retina in a novel hybrid co-culture model composed of human RPE cells and porcine/human retinal explants. The presence of RPE cells helps to maintain the retinal architecture ex vivo, as shown by Mohlin et al. when co-culturing ARPE19 cells and porcine retinal explants [23]. Furthermore, the porcine retina has morphological and functional similarities to the human retina, including its cone-rich visual streak, an avascular retina area rich in cone PR, similar to the human macula being an attractive model to study retinal function and disease [24]. Porcine retinal explants have been successfully used to study other retinal disease mechanisms, such as oxidative stress- or hypoxia-mediated damage, characteristics of several retinal degenerative diseases, including AMD [25,26]. 

In this study, we employed retinal explants obtained exclusively from the visual streak region of porcine retina to mimic as much as possible the human macula. We show that porcine retinae, but that lack porcine RPE, when exposed to human RPE cells deprived of FH undergo a faster loss of PR cells, and it appears that the rod cells are the first to undergo degeneration. Moreover, via Raman analyses, we observe that the main changes in the retina appear to be in the mitochondria and lipid composition of the co-cultured retinae upon FH loss. Furthermore, we prove that the co-culture model is also suitable for human retinal explants, and we observed a similar trend when RPE cells deprived of FH were cultured from human retinal explants from a single donor eye. Our findings highlight the importance of RPE-derived FH for retinal homeostasis and provide a valuable model for AMD research.

## 2. Materials and Methods

### 2.1. Cell Culture

Human retinal pigment epithelium (RPE) cell lines hTERT-RPE1 cells were obtained from the American Type Culture Collection (ATCC). The cells were cultured in medium containing 1:1 DMEM F12: Hams F12 (Gibco, Waltham, Massachusetts, USA) with additional supplementation of 10% fetal calf serum (FCS; Gibco, Germany), penicillin (100 U/mL) and streptomycin (100 µg/mL). These RPE cells were seeded at the rate of 100,000 cells/transwell in a 12 mm polyester transwell with 0.4 micron pore size and were allowed to grow overnight at 37 °C incubated with 5% carbon dioxide. The *CFH* gene was silenced by siRNA-mediated transfection in the RPE cells according to the manufacturer´s instructions using Viromer Blue reagent (Lipocalyx, Saale, Germany). The siRNA (IDT technologies, Leuven, Belgium) mixture was prepared using three different silencing RNAs specific for *CFH* (siCFH) and one siRNA for negative control (siNeg) in the Viromer Blue buffer. After 24 h of silencing, the media was changed, and the cells were used for co-culture.

### 2.2. Porcine Retinal Explants

The porcine eyes were obtained from the local slaughterhouse. The local institutional ethics committee approved all the experiments conducted with porcine retinal explants. The explants were prepared from 6-month-old pigs weighing on an average 100 kg within 2–3 h of enucleation as previously described [27]. After electrocution euthanization, the enucleated eyes were transported in ice directly to the research facility. The eyes were immersed in 70% ethanol, following which the muscle tissue was removed using sterile Westcott scissors in a laminar airflow chamber. A small incision was made using a sterile blade, and the anterior segment of the eye was removed, including the cornea, lens, iris and ciliary body. Sharp forceps were used to break the vitreous free from the eyecup, and the vitreous was also removed. In the avascular zone, two cone-rich zones in the retina were identified and dissected based on the pattern of blood vessels (as shown in Figure S1). The retina was then carefully peeled off from the RPE-choroid-sclera layers and retinal explants with no porcine RPE cells were placed on the transwell containing RPE cells, with the PR side facing down toward the RPE cells. From each porcine eyecup, two cone-rich retinal explants were obtained to have an internal control for each pig: one explant was exposed to control siNeg RPE cells and one explant to siCFH RPE cells. The RPE-retina was co-cultured in medium containing 1:1 DMEMF12: Hams F12 with B27 supplement (Life Technologies), penicillin (100 U/mL) and streptomycin (100 µg/mL) for 72 h in a 37 °C incubator with 5% carbon dioxide. One medium change was performed 36 h after initiating the culture, removing only half of the consumed medium, allowing a continuous exposure to possible relevant secreted factors from the RPE cells.

### 2.3. Human Retinal Explants

The human donor eye was obtained from the human tissue biobank of the Eye Clinic Tübingen (ethic 324/2013BO1). Prior informed consent was obtained from the donor, and the anonymity of the donor was maintained. The human donor was diagnosed with uveal melanoma, and the enucleation surgery was performed at the University Hospital Tübingen in Tübingen, Germany. The human eyecup was transported to the laboratory in ice within 30 min of enucleation, and experiments were performed under local ethics committee approval 185/2021BO2. The vitreous was removed using sterile forceps. The retina was randomly dissected to obtain 6 explants, excluding the region surrounding the dissected tumor. The human retinal explants were co-cultured with negative control (siNeg) and *CFH* silenced (siCFH) hTERT-RPE1 cells grown on transwell as described above. The hTERT-RPE1 cells-human retina explants were co-cultured ex vivo in medium containing 1:1 DMEMF12: Hams F12 with B27 supplement (Life Technologies), penicillin (100 U/mL) and streptomycin (100 µg/mL) for 72 h in the same conditions described above.

### 2.4. Immunohistochemistry

The co-cultured RPE-retina were washed with 1X PBS for 5 min and fixed using 4% PFA for 45 min at room temperature. After that, a sucrose gradient was performed by incubating the samples in 10%, 20%, 30% sucrose to ensure cryopreservation of the co-cultured tissue. The tissues were then snap-frozen in liquid nitrogen using Tissue-Tek Optimal cutting temperature compound. The cryo molds were stored at −20 °C until sectioning. Cryostat 12 to 14-micron thick retina-RPE co-culture sections were obtained. The cells were then permeabilized and blocked for 1 h with 0.1% Triton X-100,10% normal goat serum and 1% bovine serum albumin in PBS. The sections were then stained overnight at 4 °C in the respective primary antibodies (anti-Rhodopsin, MAB5316 Sigma Aldrich; anti-M-Opsin, AB5405 Sigma Aldrich; anti-S-Opsin, AB5407 Sigma Aldrich; anti-Iba-1, 019-19741 Wako Chemicals; anti-GFAP, G-3893 Sigma Aldrich; anti-Bestrophin-1, AB2182 Sigma Aldrich). After overnight incubation, the sections were washed thrice with PBS to remove excess primary antibodies. The recommended concentration of secondary antibodies (Goat Anti-Rabbit Alexa Fluor 488, A-11034 and Goat Anti-Mouse Alexa Fluor 568, A-11031, Invitrogen) were added, and the sections were incubated for 1 h. The sections were washed thrice in PBS, stained with DAPI for 5 min, and mounted with Fluoromount-G solution (17984-25, Electron Microscopy Sciences, Montgomery, PA, USA). Z-stack images were obtained using a Zeiss Axio Imager Z1 ApoTome Microscope, and the images were analyzed using Zen Blue image analysis software. 

### 2.5. Image Analysis

For each sample, three sections were selected, excluding sections showing rosettes or tissue damage [6]. For each section, three different retinal regions were imaged to obtain nine images for each sample. Twelve data points were obtained for each image to obtain a uniform coverage of an entire image to calculate the overall retinal thickness, thickness of ONL and number of rows in ONL. The distribution of DAPI staining, which identify nuclei, defines whether retina structure is maintained and the retinal layers can be therefore identified. All the images were taken with uniform exposure time, and the fluorescence of all the images is also brought to uniform values within the Zen Blue software to obtain a comparable mean fluorescence intensity value. To obtain the density of M- and S-cones, 3 × 100 micron regions were selected in each image, and the average of 9 × 3 = 27 data points was considered to be the number of cones in 100 µm. As most of the cones are positioned on the outermost layer of the ONL in healthy conditions, displaced cones were counted as immunostained nuclei found three rows above the outermost layer of the ONL. 

### 2.6. TUNEL Assay

TUNEL assay was used as a marker of cell death and was performed using an in situ cell detah detction kit conjugated with fluorescin isothiocyanate (Roche, Basel, Switzerland). Section were stained with DAPI for 5 min, and mounted with Fluoromount-G solution (17984-25, Electron Microscopy Sciences, Montgomery, PA, USA).

### 2.7. Hematoxylin and Eosin Staining 

Retinal sections were pre-rinsed in PBS and then dipped in Harris Hematoxylin for 5 min. Following washes in ddH_2_O, slides were dipped in acid alcohol (70% ethanol 1% HCl), washed in ddH_2_O and then dipped in Eosin-Phloxyine. Slides were then dehydrated in a series of 95% ethanol and 100% ethanol followed by incubation in xylene. Once the slides were dried, mounting medium (Entellan, Merck Milipore, Darmstadt, Germany) was applied and the slides were prepared for imaging.

### 2.8. Raman Microspectroscopy

Cryosections obtained from sample preprocessing for immunohistochemistry were thawed, rinsed with PBS and kept hydrated during the whole measurement. Raman images were recorded using a WiTec 300R alpha Raman microspectroscope (WiTec GmbH, Ulm, Germany), equipped with a green laser (532 nm), a 63× water dipping objective (Carl Zeiss AG, Jena, Germany) and a spectrograph with a 600 g/mm grating and a CCD camera. Spectral maps were acquired as 100 × 300 µm cross-sections with a pixel resolution of 1 × 1 µm, a laser power of 50 mW and an integration time of 0.1 s/spectrum. In addition, a reference spectrum of docosahexaenoic acid (DHA, #53171, Sigma-Aldrich, St. Louis, MI, USA) was generated as an average spectrum from spectral maps of the pure substance (1 × 1 µm spatial resolution, 0.1 s integration time, 50 mW laser power).

### 2.9. Raman Data Analysis

Before further analysis, the acquired spectral maps were preprocessed in the WiTec *Project Five* Software (5.4, WiTec GmbH). Data underwent cosmic ray removal, were baseline corrected (shape algorithm) and normalized (area to 1). True component analysis (TCA) was applied to generate false color-coded intensity distribution heatmaps of major tissue structures as previously described [28]. For quantitative analysis, intensity heatmaps were extracted for each component and mean GVIs were calculated in ImageJ and normalized to the cell number. Analyses were focused on the ONL region. Three spectral maps per sample was analyzed.

Furthermore, single spectra (*n* = 100/image) were extracted from the lipid-assigned regions for in-depth analysis of the molecular composition via principal component analysis (PCA) performed in The Unscambler X (Camo Analytics AS, Oslo, Norway). The application of PCA for the analysis of spectral data were described elsewhere [29]. In brief, principal component (PC) score values were compared between the two groups to identify differences in their molecular composition. The underlying molecular information can be retrieved from the corresponding PC loadings plot, indicating relevant spectral features as positive or negative peaks.

### 2.10. RNA Isolation, cDNA Synthesis and qRTPCR

RPE cells pellets were collected, and RNA was extracted using Tri-Fast reagent according to the manufacturer instructions. The concentration of the isolated RNA samples was assessed using a Nanodrop spectrophotometer. cDNA synthesis was performed via reverse-transcription of 2 µg of RNA using M-MLV Reverse Transcriptase (Promega (Madison, WI, USA)). cDNA was used to analyze silencing efficiency by qRT-PCR employing iTaq Universal SYBR Green Supermix (Bio-Rad Laboratories, USA) along with gene-specific forward and reverse primers (10 μM, RPLP0 fw 5′-GGA GAA ACTGCT GCC TCA TATC-3, RPLP0 rev 5′-CAG CAG CTGGCA CCT TAT T-3′, accession number NM_053275, ACTB fw 5′-TAA GGA GAA GCT GTG CTA CGT C-3′, ACTB rev 5′-TTC GTG GAT GCC ACA GGA C-3′, accession number NM_001101.5, *CFH* fw 5′-CTG ATC GCA AGAAAG ACC AGT A-3′, *CFH* rev 5′-TGG TAG CACTGA ACG GAA TTAG-3′, accession number NC_000001.11).

### 2.11. C3b ELISA

Based on the manufacturer’s instructions, the concentration of C3/C3b in culture supernatants were analyzed by ELISA assay (Abcam, Cambridge, UK). Samples were loaded undiluted along with standards and controls in 96 well-plates coated with specific a C3b antibody, and the assay was performed according to the manufacturer instructions. Absorbance was immediately measured at 450 nm using a Spark multimode microplate reader (Tecan, Männedorf, Switzerland). To correct optical imperfections, subtraction readings were taken at 570 nm. 

### 2.12. Statistical Analysis

The graphs were plotted as mean ± SEM using Graph Pad Prism version 8, and the statistical analysis was performed using SPSS version 2.2 and Graph Pad Prism version 8. The quantification of western Blot signals and fluorescent images was done using ImageJ or Zen Blue Image analysis software, respectively. Unpaired and paired t-test were used to assess the differences in the means between different groups. A *p*-value less than 0.05 is considered statistically significant. 

## 3. Results

### 3.1. Porcine Retinal Explants Exposed to FH-Deprived RPE Cells Shows Signs of Degeneration

Our previous work has shown that the homeostasis of RPE cells deprived of FH is strongly impaired. This study aimed to evaluate whether and how RPE cells deprived of FH affect homeostasis of the neuroretina. We used a co-culture system comprising human hTERT-RPE1 cells and porcine retinal explants obtained from the porcine visual streak cone-rich area to study the impact of those impaired RPE cells on the retina. hTERT-RPE1 cells were either silenced for the CFH gene (siCFH) or treated by an siRNA negative control (siNeg); (Figure S2B–D). Preliminary work was performed to identify the most suitable time point for both the silenced hTERT-RPE1 cells and the retinal explants (shown in Figure S2). We chose 3 days ex vivo cultures for all further experiments, because 3 days porcine retinal cultures show a minimal level of cell death, especially in the outer nuclear layer (ONL) as indicated by the TUNEL positive cells (Figure S2E). Furthermore, H&E staining (Figure S2F) shows that the retinal structure is maintained at this time point. 

**Figure 1:**
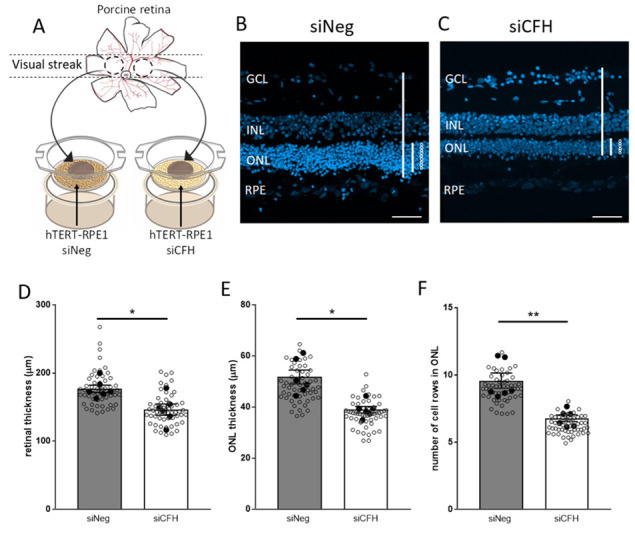
FH loss mediates changes in the co-cultured porcine retina. (**A**) Schematic representation of the co-culture system, in which two retinal explants from the visual streak of porcine donor eyes are exposed to either siNeg or siCFH hTERT-RPE1 cells for 72 h. (**B**,**C**) The porcine retinal architecture was assessed with the help of DAPI, a nuclear staining dye. Representative images of siNeg co-culture (**B**) and siCFH co-culture (**C**) are shown. Lines and circles highlight the parameters extrapolated from these images and quantified in (**D**–**F**) the overall thickness of the porcine retina (**D**), the thickness of the ONL (**E**) and the number of rows in the ONL (**F**). Abbreviations: GCL—Ganglion cell layer, INL—inner nuclear layer, ONL—outer nuclear layer, RPE—retinal pigmented epithelium cells, siNeg—silencing negative control, siCFH—silencing CFH. Mean ± SEM is shown. *N* = 6 biological replicates. * *p* ≤ 0.05; ** *p* ≤ 0.005. Scale bar—50 µm.

Retinal explants, without porcine RPE cells, were placed in direct contact with the silenced hTERT-RPE1 cells with the PR side facing the RPE cells, which mimics the in-vivo condition (Figure 1A). Porcine retinal explants co-cultured for 3 days ex vivo with FH-deprived hTERT-RPE1 cells (siCFH) exhibited signs of degeneration compared to retinal explants co-cultured with siNeg hTERT-RPE1 cells (Figure 1B,C). In detail, overall retinal thickness was reduced (176.8 ± 5.3 µm vs. 146.4 ± 8.2 µm, Figure 1D), the thickness of the outer nuclear layer (ONL) was smaller in siCFH co-cultured retinae (51.7 ± 2.7 µm vs. 38.9 ± 1.2 µm, Figure 1E) and the number of cell rows in the ONL was lower (8.4 ± 0.5 vs. 6.1 ± 0.2; Figure 1F). To ensure that the effects observed were dependent on the status of the RPE cells and not dependent on the loss of RPE cells, which could occur upon FH loss, we exposed the retinal explants to different concentrations of hTERT-RPE1 cells (Figure S3). Our results indicate that porcine retina explants co-cultured with either 50.000 or 100.000 hTERT-RPE1 cells (seeding density) displayed a similar retinal structure (seeding density also employed for siNeg-siCFH experiments). In fact, exposure of 200.000 hTERT-RPE1 cells to the explants had a negative impact on the retinal parameters investigated, and in particular, ONL thickness was significantly reduced (Figure S3). 

### 3.2. FH Loss in RPE Cells Mediates Rod Cell Degeneration in the Co-Cultured Porcine Retina

**Figure 2:**
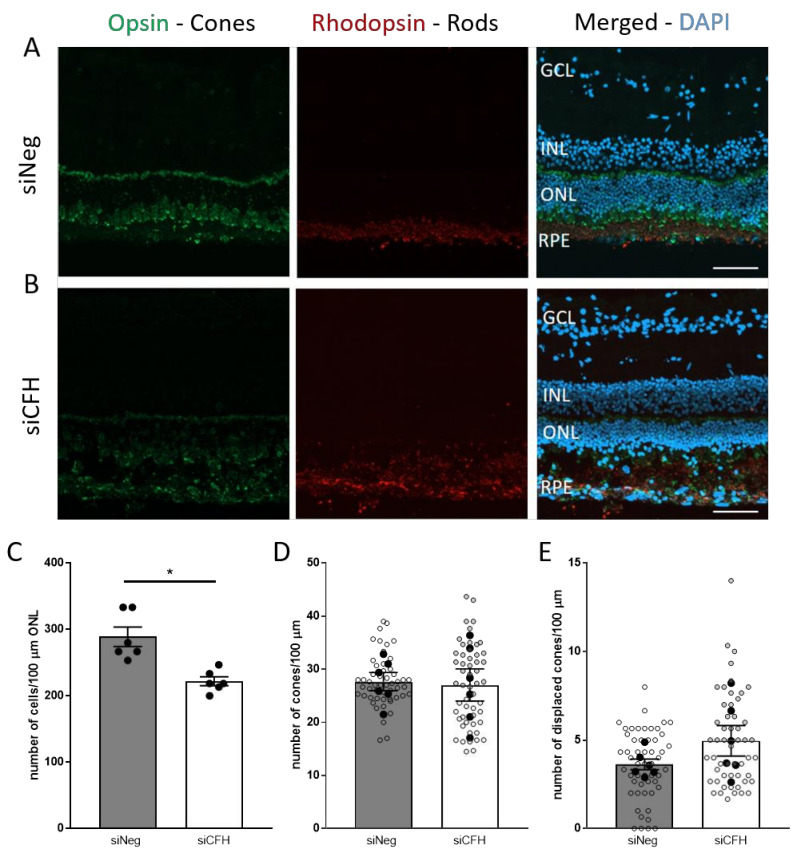
Effect of FH loss in RPE cells on porcine PR in co-culture. (**A**,**B**) Retinal explants from the visual streak of porcine donor eyes are exposed to either siNeg (**A**) or siCFH (**B**) hTERT-RPE1 cells for 72 h. Explants cryosections were stained for Opsin (green) and Rhodopsin (red) to differentiate between cones and rods, and DAPI was used as counterstaining for nuclei. (**C**–**E**) Quantification of retinal parameters extrapolated from A-B: number of cells in ONL (**C**), number of cones in ONL (**D**) and number of displaced coned (**E**). Abbreviations: GCL—Ganglion cell layer, INL—inner nuclear layer, ONL—outer nuclear layer, RPE—retinal pigmented epithelium cells, siNeg—silencing negative control, si*CFH*—silencing *CFH*. Mean ± SEM is shown. *N* = 6 biological replicates. * *p* ≤ 0.05. Scale bar—50 µm.

Next, we focused on investigating which cell type was mainly affected by FH loss in RPE cells. Porcine rod and cone PR were immune-labeled with rhodopsin and opsin antibodies, respectively, after being in co-culture with either siNeg or siCFH hTERT-RPE1 transfected cells for 3 days (Figure 2A,B). Cell counting revealed a significant reduction in the total number of cells in the ONL in siCFH condition, indicating a loss of PR cells when the retinae were in contact with CFH deficient RPE cells (253.3 ± 14.5 cells vs. 200 ± 6.6 cells, Figure 2C). To understand whether rod or cone PR cells are degenerating first, we analyzed the specific rhodopsin and opsin staining. We did not observe any quantitative reduction in total M-cones (Figure 2D), indicating that a loss of rods accounts for the reduced number of cells in ONL. Although the number of M-opsin positive cells (Figure 2D) were similar in both groups, both groups show a certain level of opsin mislocalization, a sign of unhealthy cones, which has to be expected and taken into consideration in an ex vivo explant model. However, the distributions of M-cones was even more affected in the absence of FH in the RPE cells, and more displaced cones were counted when the retinae were co-cultured with siCFH hTERT-RPE1 cells (Figure 2E). 

Our previous study shows that FH deprivation in RPE cells leads to elevated secreted levels of C3 [22]. To assess whether C3 directly mediates retinal damage, we supplemented the culture medium with purified C3. Exogenous addition of purified C3 to the porcine retina co-cultured with either siCFH or siNeg hTERT-RPE1 cells had no impact on the retinae (Figure S4). Moreover, we evaluated whether the addition of purified FH could overcome the absence of endogenous FH in RPE cells. Exogenous addition of purified FH protein to the co-culture media did not affect the porcine retina when compared to samples without additional FH (Figure S4). Our data indicate that exogenously present C3 or FH does not impair neuroretinal homeostasis, but that this is due to endogenous changes within the RPE exposed to the retina. 

Moreover, RPE cells deprived of FH show an increase in inflammatory cytokines production [22]. Since inflammation is a sign of degeneration [30], we assessed the total levels of microglia cells within the retinal explants and their migratory patterns, since microglia cells provide different effects depending on their location in the retina [30]. After three days of co-culture, we evaluated the number of Iba1 positive cells as an indicator of microglial cells in retinal explants co-cultured with both siNeg and siCFH hTERT-RPE1 cells (Figure S5) and we did not find any difference in the total number of cells. Moreover, when counting the number of microglia cells in the different regions of the retina, we did not observe any difference in any of the retinal layers (Figure S5). 

### 3.3. RPE Cells Deprived of FH Have a Negative Impact on the Mitochondria of Cells in the ONL 

**Figure 3:**
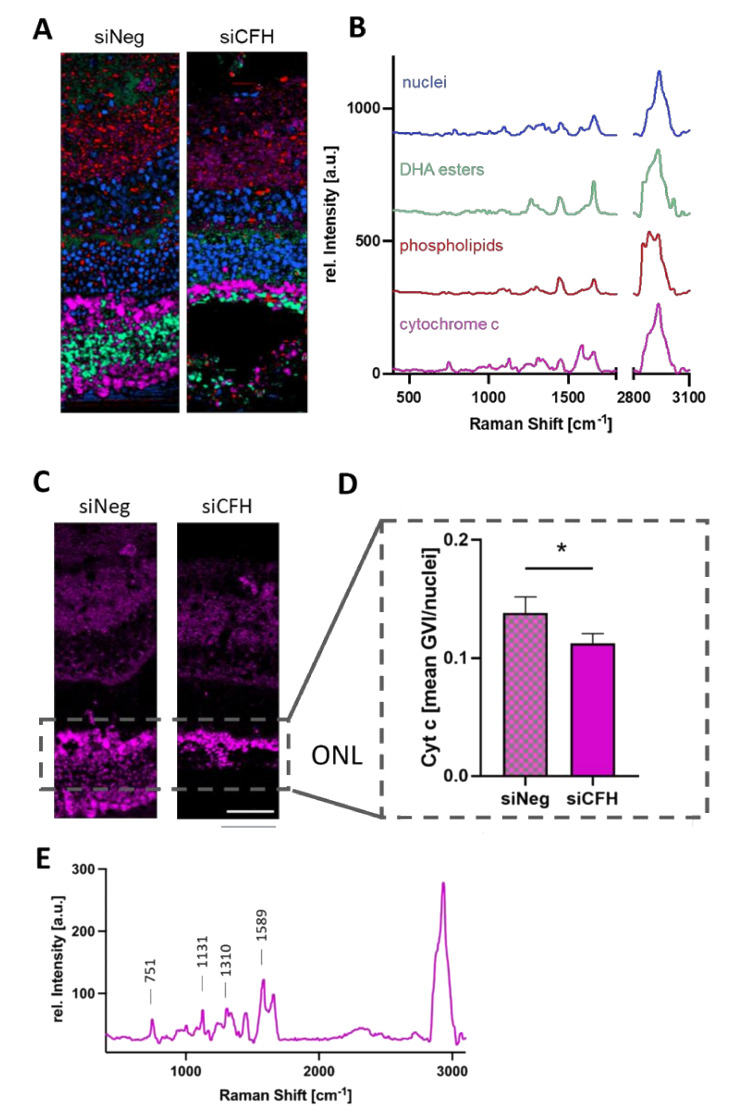
(**A**,**B**) True component analysis (TCA) was performed on the spectral maps of siNeg and siCFH samples to generate intensity distribution heatmaps (**A**). According to different spectral signatures (**B**), pixels assigned to nuclei (blue), cytochrome c (pink) and two different lipid components (red and light green) could be identified and localized. (**C**,**D**) Quantitative analysis of the mitochondrial activity of the ONL region based on the cytochrome c intensity distribution images (**C**) and calculation of the mean GVI/cell (**D**). (**E**) Raman signature of the mitochondrial activity TCA component. ONL—outer nuclear layer, siNeg—silencing negative control, siCFH—silencing CFH. Data are presented as mean ± SD. *N* = 3 biological replicates. * *p* ≤ 0.05; Scale bar—50 µm.

To understand the type of damage that RPE cells deprived of FH cause in the retina, we performed Raman imaging for marker-independent and molecular-sensitive analyses. Raman microspectroscopy allows the generation of a specific molecular fingerprint based on the assignment of spectral bands to distinct molecular groups. Although Raman analyses does not give information on the ultrastructural properties, it enables identifying and localizing different subcellular structures in the samples (Figure 3A). True component analysis (TCA) allowed distinguishing nuclei, mitochondria and two different groups of lipids according to their specific spectral signatures (Figure 3B). Nuclei showed specific DNA-related bands at 792, 1098 and 1580 cm^−1^, mitochondria were characterized by their cytochrome C signals at 750, 1130 and 1589 cm^−1^ and the lipid components showed peaks at 720, 1450, 1655 and 2850 cm^−1^ correlating with phospholipids and fatty acids. A detailed overview of all peaks and their molecular assignments are shown in Table S1. First, the mitochondria signal was analyzed in detail. Since the ONL is the layer of the neuroretina affected in AMD, we specifically quantified cytochrome c signal intensities within this region (Figure 3C) and demonstrated a loss in cytochrome c signal in the ONL of the co-culture samples with the siCFH RPE cells (Figure 3D). The identified cytochrome c spectrum (Figure 3E) corresponds to the signature of the reduced, bound form found in healthy cells and has a different Raman signature compared to oxidized cytochrome c [31,32], which is released into the cytosol upon apoptosis. The reduction of cytochrome c in the mitochondria in the ONL of the FH-deprived group indicates lower mitochondrial activity and might be a consequence of oxidative stress.

### 3.4. Retinae Cultured with siCFH RPE Cells Showed Signs of Lipid Oxidation in the ONL

**Figure 4:**
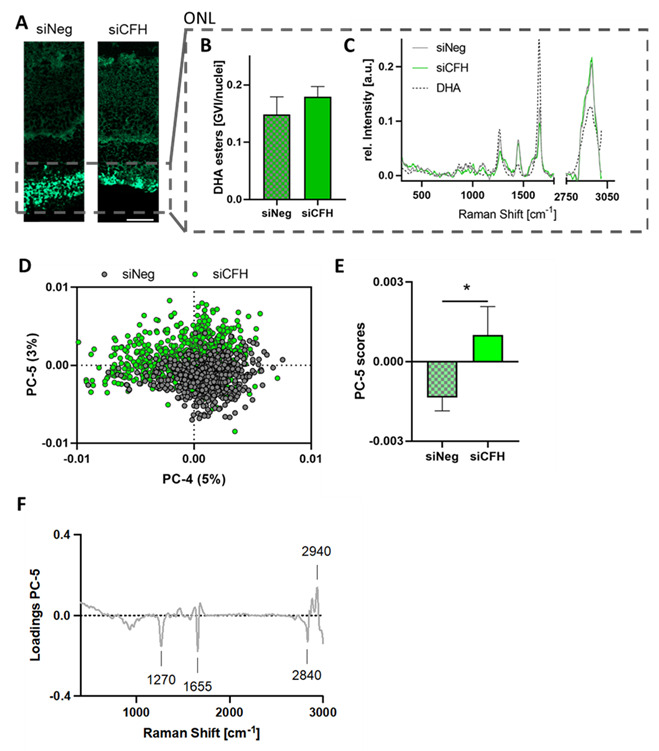
(**A**) TCA intensity distribution heatmaps of the first lipid feature showed a dominant localization in the ONL region. (**B**) Quantification of lipid signal/cell in the ONL. (**C**) Overlay of a DHA reference spectrum (black, dashed) with the average siCFH (green) and siNeg (gray) spectra from the lipids in the ONL region. (**D**–**F**) Principal component analysis (PCA) of single spectra (*n* = 100 per sample) extracted from siNeg (gray) and siCFH (green) ONL regions demonstrates a clustering in the PCA scores plot (**D**) with a significant difference described by PC-5 (**E**). The most relevant spectral signatures are demonstrated in the loadings plot (**F**). Data are represented as mean ± SD, *n* = 3, * *p* ≤ 0.05, scale bar—50 µm.

Raman image analysis via TCA identified two lipid components that showed different distribution patterns within the retina (Figure 3A). Whereas the first component (green) was predominantly localized in the ONL, the second lipid signal (red) was associated with the inner retina. The ONL assigned lipids were quantified based on their signal intensities (Figure 4A,B) and showed no significant difference between both groups. Average spectra from siCFH and siNeg were compared to a reference spectrum of docosahexaenoic acid (DHA), one of the most abundant phospholipids in the outer ONL [33], to identify the lipid subtype. The spectral overlay (Figure 4C) demonstrated similar spectral signatures between DHA and the ONL lipids. For in-depth analysis and in addition to quantitative analysis, qualitative analysis via PCA was performed to investigate if changes in lipid composition are induced upon FH deprivation. Comparison of the single spectra in the PCA scores plot (Figure 4D) and analysis of the average PC score values (Figure 4E) demonstrated a significant shift in the spectral signatures of both groups described by PC-5. The assigned spectral changes were exploited as negative (siNeg) or positive (siCFH) peaks in the corresponding PC-5 loadings plot (Figure 4F).

In comparison to siCFH samples, the control samples had more intense bands at 1270 and 1655 cm^−1^ and a higher 2850 cm^−1^ signal, whereas the siCFH group was characterized by an increase of the 2940 cm^−1^ band. The identified spectral features correspond to C-H and C=C bonds in lipid molecules [34] and have been reported before in the context of lipid oxidation, where oxidative processes have been linked to decreases in the 1270 cm^−1^ and 2840 cm^−1^ region and to an increase of signal intensity at the 2940 cm^−1^ band [35,36]. Thus, these findings correlate with our observations and indicate the relevance of lipid oxidation in DHA esters as one of the destructive processes induced by siCFH RPE cells.

Furthermore, we analyzed the Raman images of the second lipid component, which was mainly localized in the inner retina and contained phospholipid-assigned spectral features (Figure S6B). There was no quantitative difference detected in the lipid signal intensities of both groups (Figure S6A,C), and no significant structural alteration occurred, as demonstrated by no separation in any of the calculated PCs (Figure S6D). 

These results lead to the conclusion that the FH-deprived RPE cells do impact the lipids in the ONL—most likely by oxidative processes on the DHA esters—but do not induce further alterations on the phospholipids of the inner retina layers.

### 3.5. FH Loss in RPE Cells Leads to Degeneration in Human Retinal Explants 

**Figure 5:**
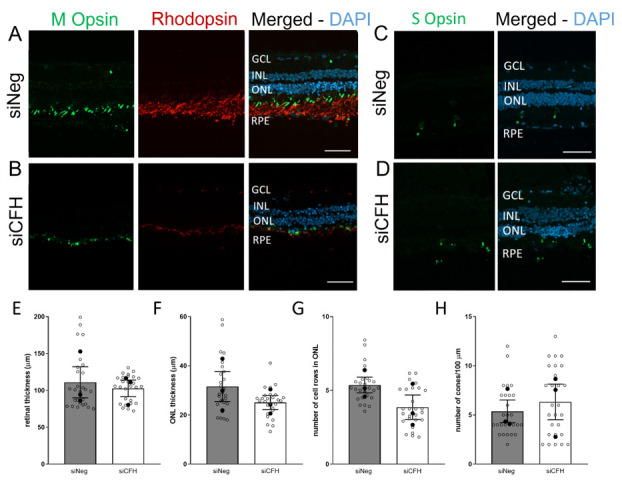
Changes mediated by FH loss in RPE cells in the co-cultured human retina. Human retinal explants from a single donor eye are exposed to either siNeg (**A**–**C**) or siCFH (**B**–**D**) hTERT-RPE1 cells for 72 h. (**A**,**B**) Explants cryosections were stained for M-Opsin (green) and Rhodopsin (red) to differentiate between cones and rods, and DAPI was used as counterstaining for nuclei. (**C**,**D**) Explants cryosections were stained for S-Opsin (green), and DAPI was used as counterstaining for nuclei to identify S-cones. (**E**–**H**) Quantification of retinal parameters extrapolated from (**A**–**D**): retinal thickness (**E**), ONL thickness (**F**), number of cell rows in ONL (**G**) and number of cones in ONL (**H**). Abbreviations: GCL—Ganglion cell layer, INL—inner nuclear layer, ONL—outer nuclear layer, RPE—retinal pigmented epithelium cells, siNeg—silencing negative control, si*CFH*—silencing *CFH*. Mean ± SEM is shown. *N* = 3 biological replicates from a single donor eye. Scale bar—50 µm.

Ultimately, we investigated whether the effects observed in porcine retinal explants are conserved in the human retina in response to FH-deprived RPE cells. Due to the scarce availability of human eye donors, explants from one donor were collected and exposed to either siNeg or siCFH hTERT-RPE1 cells for 72 h (Figure 5A–D), and the same retinal parameters were analyzed as for the porcine retinal explants. First, we confirmed that a co-culture system including human RPE cells and human retinal explants from different sources is possible. However, we did not observe any changes in retinal thickness (Figure 5E). Moreover, ONL thickness of the retinal explants co-cultured with siCFH hTERT-RPE1 cells was slightly reduced compared to siNeg controls (21.9 ± 6.1. µm vs. 20.6 ± 2.8 µm; Figure 5F).

Similarly, the number of ONL cell rows in the retina co-cultured with siCFH hTERT-RPE1 cells was lower (4.6 ± 0.5 vs. 2.6 ± 0.8, Figure 5G). Although these reductions in the number of rows in ONL and the ONL thickness are not statistically significant, this decline might be indicative of a loss of PR, most likely of rod PR since also in the case of human retinal explants, the number of cones was not affected at this time point (Figure 5H). Additionally, the hypothesis that rod degeneration precedes cones degeneration in this model is supported by the observation that rod outer segments are strikingly smaller in retinae co-cultured with siCFH than siNeg hTERT-RPE1 cells (Figure 5A,B).

## 4. Discussion

AMD is a complex multifactorial disease whose etiology and pathology are not yet fully understood [37]. As analysis of pathological changes in the living organism appears difficult, ex vivo retinal experimental models are needed. Here, we present a human RPE- porcine retina hybrid co-culture model that mimics some aspects of AMD and aids in understanding the interdependency of RPE cells and the neuroretina. The first advantage of this model is the use of the avascular cone-rich zone in the porcine eye, which is more similar to the para-foveal cone-rich region of the human eye than commonly used rodent models [6]. Secondly, this organotypic culture preserves the complex cell-cell interaction of the retina, including glia-neuron and RPE-retina interactions, which are important for maintaining retinal structure and function ex vivo. Moreover, the porcine eyes that are more readily available as a by-product of the meat industry can be used for larger screening, and validation can be performed using human donor eyes, which are more rarely available. Indeed, we demonstrate that our model is also suitable for human RPE-human retinal explant co-culture. 

The model is not intended to replicate AMD fully, but it is designed to answer specific questions concerning AMD pathology. In particular, our goal was to create a model to assess the impact of different RPE cells on the neuroretina. The porcine retina explantation offers the chance to detach the neuroretina from the RPE cells quite easily, with no major mechanical force. In this way, we were able to expose a retina with no RPE to a previously prepared RPE cells of our choice. However, as with any explants model, the main limitation lies on the degree of degeneration that the retinal explants undergo once in culture due the axotomy of the optic nerve and culture conditions [38] and for those reasons, the time that retinal explants could be cultured is variable. In our study, the time point was limited to three days in vitro and further effort will be employed to improve culture conditions to allow longer time points. Considering that the RPE cell component of the model can be generated from patient-specific iPSC, and can be genetically manipulated or exposed to AMD-related stressors prior to co-culture, this model is flexible and can advance our understanding on how healthy/diseased RPE affects the neuroretina. 

In this case, the diseased RPE cells for this study were molecularly manipulated by siRNA-based down-regulation of FH, a known complement system inhibitor and one of the main genetic risk factors for AMD. In fact, the Y402H polymorphism in the FH protein, which leads to a decrease of its activity, predisposes for AMD. As a consequence of its multi-functional properties, its specific role within AMD pathogenesis is not understood. [4]. When secreted, FH acts as an inhibitor of the alternative complement pathway. The FH 402H polymorphism impairs this activity, resulting in higher complement activation and inflammation, which contributes to pathological features of AMD [4]. Recently an additional intracellular role for FH, independent from complement regulation has been shown in different cell types, including RPE cells [18,19]. In our previous studies [18,22], we have shown that loss of intracellular FH in hTERT-RPE1 cells via *CFH* silencing affects several AMD-relevant features and pathways in RPE cells in a similar way as the *CFH* 402H variant in iPS-RPE cells [20,21]. In particular, *CFH* silenced RPE cells present decreased mitochondrial respiration and glycolysis, indicating a general reduction in metabolic capacity. Moreover, *CFH* silenced RPE cells show increased vulnerability to oxidative stress and an increase in oxidized lipids content, both features found in AMD. Therefore, a goal of this study was to investigate whether FH deprivation in RPE cells is sufficient to have detrimental effects on the neuroretina, independently from systemic complement sources. Additionally, the aim was to identify the main cell types affected in the neuroretina and the main changes in the ONL, as photoreceptors are the main neuroretinal cell-type affected in AMD.

First, we examined the effects that RPE deprived of FH have on the structure of the co-cultured retina. We observed a reduction in all three parameters—retinal thickness, ONL thickness and the number of rows in ONL, indicating that RPE cells deprived of FH are less able to support the co-cultured porcine retina therefore inducing retinal degeneration. In a *CFH* deficient mouse model, a similar phenomenon was reported in terms of PR loss in aged mice. It has been postulated that this could be due to RPE dysfunction and retinal stress [39]. For another mouse model of retinal degeneration, where RPE damage was induced by deletion of Superoxide Dismutase 2 (*Sod2*) [40], it was reported that the damage to the RPE cells precedes and induces PR loss. The reduction in thickness and the number of cell rows in the ONL in our model clearly indicates a loss of PR. However, as for AMD, it is under debate whether rods or cones degenerate first. Rods and cones differ in their metabolism [41], which contributes to differential susceptibility to different stress conditions [42,43]. These differences in metabolism may explain why *CFH* AMD variants, causing RPE metabolic damage, cause the PR cells in the macula to be more affected than the PR in the periphery. Moreover, in the macula region, the RPE cells have the sole responsibility for nutrient supply, while in the periphery a double vascular system is in place [44], therefore RPE dysfunction in the macula may lead to a worse outcome. In our study, we found that after three days in co-culture, FH deprivation in RPE cells induced loss of only rod PRs since the number of cones was not significantly changed. A similar trend was observed in AMD patients, where rod degeneration precedes cone degeneration in the para-foveal regions in the early stages of AMD [45]. This result implies that the rod dominant retinal regions are more susceptible to early degenerative changes in this co-culture model. However, in some cases, the cone nuclei, which are aligned in one single row at the most outer nuclear layer, appear displaced. Displacement of cone nuclei towards the outer plexiform layer of the retina has been previously described in another model of retinal degeneration as well as in post mortem donor eyes from aged individuals and AMD patients [46], where it has been suggested that displaced cones, presumably losing their synaptic contacts do not immediately undergo cell death; but lose their functionality.

We demonstrate that RPE cells silenced for FH cause PR degeneration. As the next step, we aimed to identify the mechanism by which this damage occurs. As a complement regulator, FH is part of the innate immune system, and FH-deprived cells modify the microenvironment toward an inflammatory milieu [22]. One of the major contributors to neuro-inflammation are the retinal microglial cells [30,47]. Upon excessive activation of microglial cells, the retina undergoes chronic inflammation, leading to retinal degeneration [48]. Our results showed that the loss of FH in the RPE cells did not impact the number and activation of the microglial cells. This result suggests a glial cell-independent inflammatory response, as reported in other neurodegenerative diseases [49]. Previous studies on retinal degeneration have suggested that PR loss can also be dependent on modification of Müller cell structure and metabolism [50]. However, no significant differences were observed in Müller cell activation, implying the absence of reactive gliosis in this model of FH loss induced retinal degeneration. Most importantly, we show that exogenous application of FH to the co-culture media does not rescue the neuroretina and that additional C3 does not exacerbate the effects of FH loss or cause any damage in control cultures. These findings support the hypothesis that the neurodegeneration seen here is due to intracellular changes caused by loss of FH within the RPE cells. 

RPE cells are structurally and metabolically contributing to retinal homeostasis [12]. RPE cells are responsible for nutrient transfer from the choroid to the retina. Any impairment in RPE metabolism would affect the amount or type of nutrients that reach the retina, leading to alteration of retinal metabolism [17]. Moreover, given its high metablic activity, the retina consists of a highly oxidative natural environment. RPE cells buffer this and therefore protect the retina from oxidative stress. In the context of AMD, several studies reported impairment in metabolic homeostasis and oxidative balance. We found alterations in mitochondria metabolism and oxidative stress tolerance in the RPE cells deprived of FH used in this study. We observed a reduction in the mitochondrial protein cytochrome c, specifically in its reduced form. The reduction of cytochrome c levels has been previously described in cardiomyocytes after treatment with H_2_O_2_, correlating with oxidative stress [32]. Another consequence of oxidative stress in the retina is lipid peroxidation [51]. Of note, lipid peroxidation products are one of the main components of drusen and FH dysregulation, and the FH 402H variant has already been associated with increased lipid peroxidation. Phospholipids are the main component of cellular membranes, and DHA is the most abundant polyunsaturated fatty acid (PUFA) of the retina. DHA fulfils critical functions in providing a conducive membrane environment for rhodopsin-based phototransduction with single-photon sensitivity, stabilization of PR outer segment membrane rim curvature, and fast synaptic vesicle fusion. DHA is also a main fatty acid chain of brain cardiolipin [52], an essential phospholipid constituent of the inner mitochondrial membranes. More than one-third of its acyl chains carry at least one DHA chain and because of its wedge-like shape, cardiolipin stabilizes mitochondrial cristae shape [53,54]. Therefore, oxidation of DHA has detrimental consequences on the neuroretina. When analyzing the lipid content via Raman in retinae cultured with FH-deprived cells, we observed indeed an increase in the oxidized fraction of DHA esters in the outer and inner segments of the ONL (PR), which could indicate mitochondria and outer segment damage. 

In summary, we developed a human RPE-porcine retina hybrid co-culture model of PR degeneration induced by a lack of FH in the RPE cells. Moreover, we provide evidence that the mechanism for this degeneration is likely dependent on novel regulatory intracellular activities of FH in RPE cells and is not due to systemic activation of the alternative complement pathway. For instance, our data show that the retina, particularly the ONL, is affected at the level of mitochondria stability and oxidative stress balance due to perturbations in the RPE. This may, at least in part, explain, why rods especially degenerate in AMD prior to the occurrence of geographic atrophy. 

In general, our hybrid co-culture can serve as a preclinical model to study the pathogenesis of AMD, where the RPE component can be modified depending on the scientific question. For example, retinal explants can be exposed to iPSC-RPE carrying AMD high-risk genetic polymorphisms to feature individual patient-specific endophenotypes. Furthermore, this co-culture model is amenable to the introduction of additional cell types, relevant to AMD, such as infiltrating immune cells in the lower chamber of the transwell system or endothelial cells on either side of the membrane. Finally, our model can be used to screen potential therapeutic drugs to target AMD at the early stages of disease progression.

## Supplementary Materials

The following are available online at https://www.mdpi.com/article/10.3390/biom11111621/s1, Figure S1: Preparation of porcine retinal explants for co-culture with RPE cells. Figure S2: Characterization of hTERT-RPE1 cells and retinal explants used in the co-culture model. Figure S3: Effects of RPE cells seeding density on the co-cultured porcine retinae. Figure S4: Exogenous FH or C3 does not rescue or exacerbate the effects of FH loss in RPE cells on the co-cultured porcine retinae. Figure S5: FH loss in RPE cells does not affect inflammatory cells on the co-cultured porcine retinae. Figure S6: siCFH RPEs do not have an impact on the lipid expression and composition in the inner retina [55,56,57]. Table S1: Overview of Raman peaks and their molecular assignments.
