# Supplementary material for: Complement Factor H Loss in RPE Cells Causes Retinal Degeneration in a Human RPE-Porcine Retinal Explant Co-Culture Model
Source: Biomolecules. 2021 Nov 3;11(11):1621. doi: 10.3390/biom11111621 (PMC8615889; doi:10.3390/biom11111621)
Supplement: Supplementary file 1 [file biomolecules-11-01621-s001.zip › biomolecules-1424013-supplementary.pdf]

biomolecules

MDPI

Article

Supplementary material: Complement Factor H loss in RPE cells causes retinal degeneration in a human RPE-porcine retinal explant co-culture model

Angela Armento[1*], Aparna Murali[1,2*], Julia Marzi[3,4,5], Ana C Almansa-Garcia[1], Blanca Arango-Gonzalez[1], Ellen Kilger[1], Simon J Clark[1,8], Katja Schenke-Layland[3,4,5,6], Charmaine A Ramlogan-Steel[2,7], Jason C Steel[2,7], Marius Ueffing[1]

[1]Institute for Ophthalmic Research, Department for Ophthalmology, Tübingen, Germany.

[2]Faculty of Medicine, University of Queensland, Australia.

[3] Institute of Biomedical Engineering, Department for Medical Technologies and Regenerative Medicine, Eberhard Karls University Tübingen, Tübingen, Germany. [4]NMI Natural and Medical Sciences Institute at the University of Tübingen, Reutlingen, Germany.

[5]Cluster of Excellence iFIT (EXC 2180) "Image-Guided and Functionally Instructed Tumor Therapies", Eberhard Karls University Tübingen, Tübingen, Germany

[6]Department of Medicine/Cardiology, Cardiovascular Research Laboratories, David Geffen School of Medicine at University of California, Los Angeles, CA, USA

[7]School of Health, Medical and Applied Sciences, Central Queensland University, Australia

[8] Lydia Becker Institute of Immunology and Inflammation, Faculty of Biology, Medicine and Health, University of Manchester, UK

* Both authors share equal first authorship; correspondence should be addressed to:

Angela Armento, PhD, angela.armento@uni-tuebingen.de, Phone: +49 7071 29 84953; Marius Ueffing, PhD, marius.ueffing@uni-tuebingen.de. Institute for Ophthalmic Research, Department for Ophthalmology, Eberhard Karls University of Tübingen, Tübingen, Baden-Württemberg, 72076, Germany

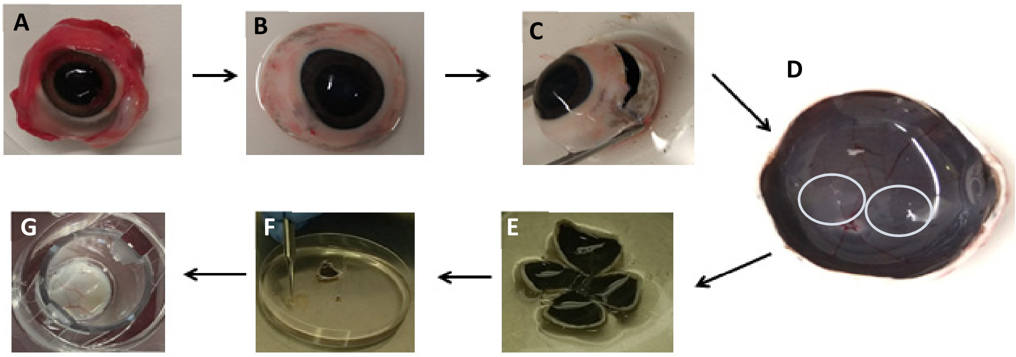

Figure S1: Preparation of porcine retinal explants for co-culture with RPE cells. **A.** Porcine eyecups were obtained from the slaughter house. **B.** Porcine eyecups after muscles and optic nerve removal. **C.** A small incision at about 5mm from the iris. **D.** The anterior segment of the eyecup including cornea, iris, lens, and ciliary body were removed. The avascular cone-rich region of the retina was identified as indicated by white circles. **E.** Flattened eyecup. **F.** Retinal fragments gently peeled off from the RPE-choroid-sclera layers. **G.** Retinal explant placed on the transwell containing the RPE cells for co-culture.

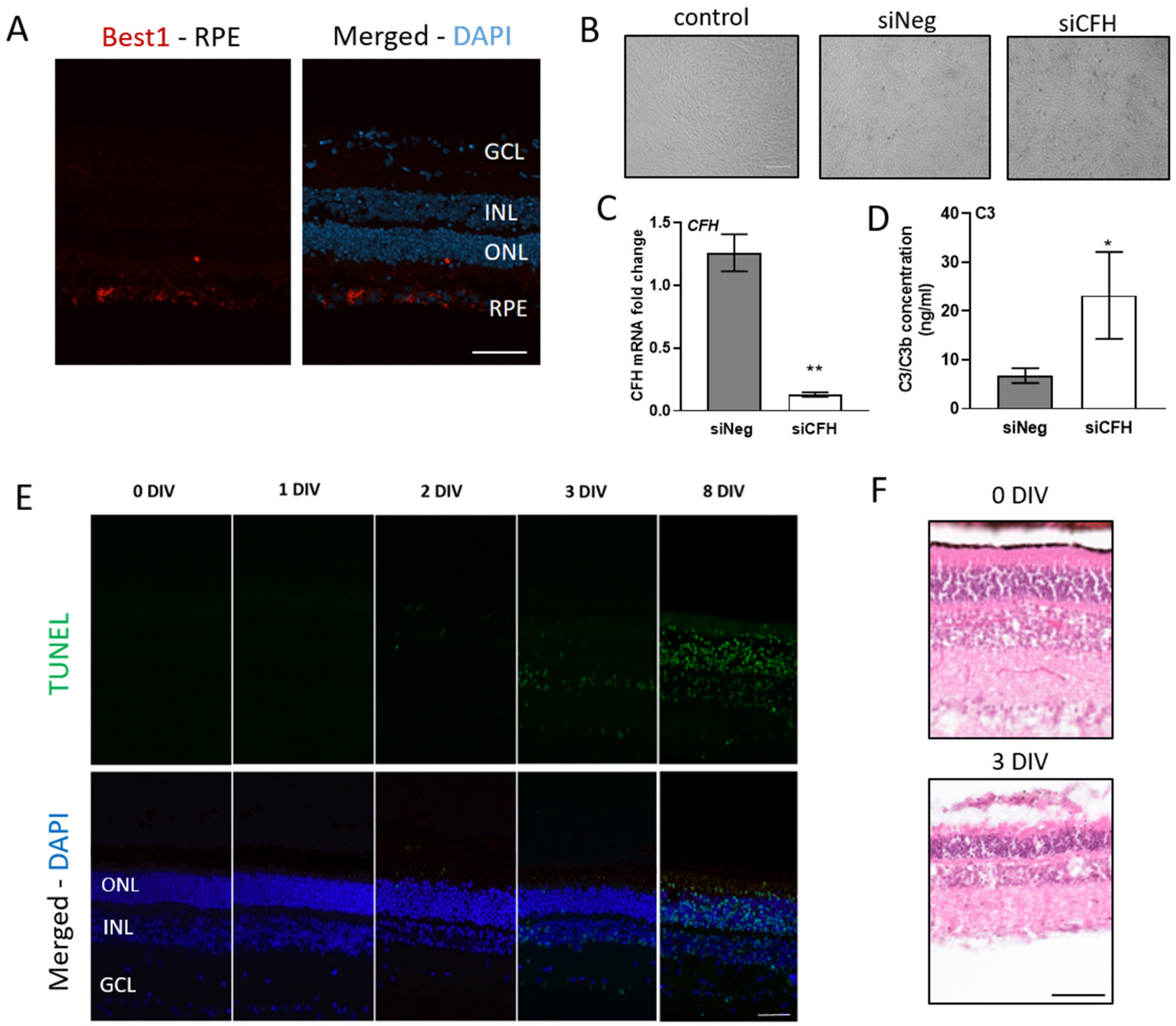

Figure S2: Characterization of hTERT-RPE1 cells and retinal explants used in the co-culture model. **A** porcine retinal explant in co-culture with hTERT-RPE1 cells. Cryosections were stained for RPE marker Best1 (red) and DAPI staining for nuclei. **B** Photographs of control hTERT-RPE1 cells and hTERT-RPE1 cells silenced with siNeg or siCFH siRNA.**C-D** Cell pellets and cell culture supernatants from hTERT-RPE1 cells siNeg and siCFH were collected after 72 hours. **C** Evaluation of *CFH* expression after 72 hours by qRT-PCR analyses. Data are normalized to the housekeeping genes RPLP0 and ACTB using Δ ΔCt methods. SEM is shown, n=4. **D** C3/C3b ELISA analyses of cell culture supernatants of hTERT-RPE1 cells. SEM is shown, n=4. E Tunel (green) and Dapi (blue) staining of porcine retinal explants at different time points in culture (DIV-days *in vitro*). **F** H&E staining of retinal sample 0 DIV and 3 DIV.

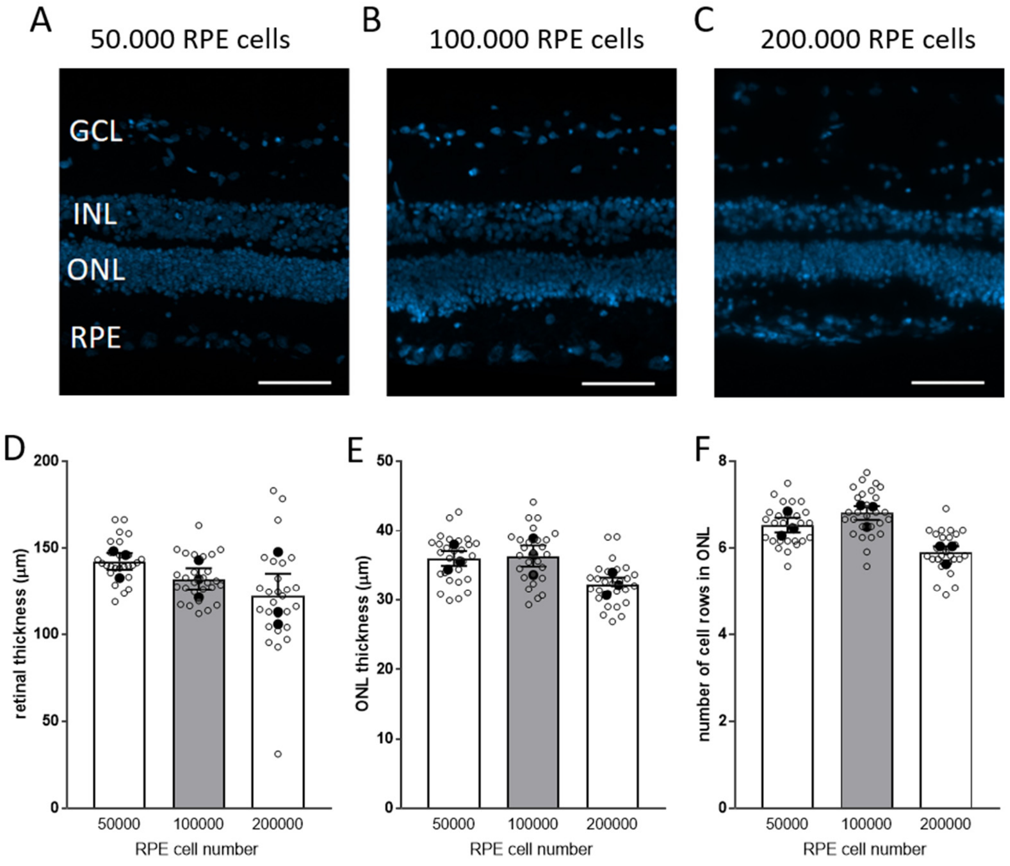

Figure S3: Effects of RPE cells seeding density on the co-cultured porcine retinae. Retinal explants were exposed to hTERT-RPE1 cells seeded at increasing cell density: **A** 50.000 cells, **B** 100.000 cells and **C** 200.000 cells. Retinal architecture parameters were assessed with the help of DAPI staining for nuclei. Quantification of the parameters are shown in D-F: retinal thickness (D), ONL thickness (E), number of cell rows in the ONL (F). Mean ± SEM is shown. N=3.

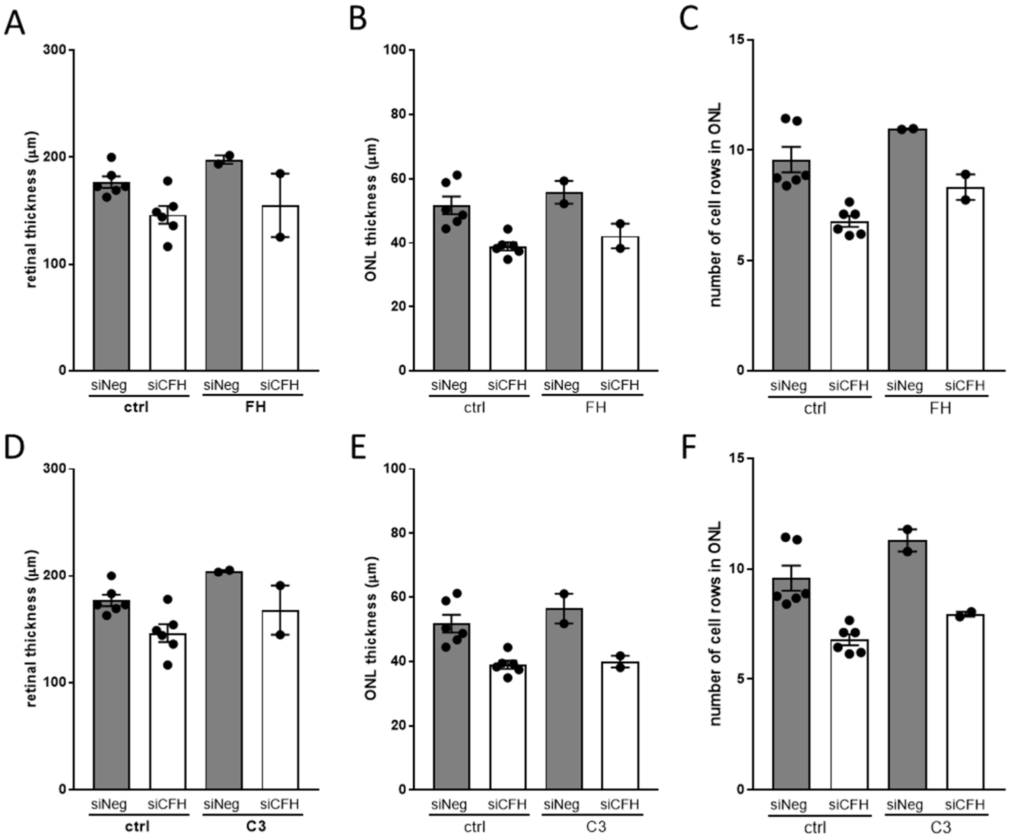

Figure S4: Exogenous FH or C3 does not rescue or exacerbate the effects of FH loss in RPE cells on the co-cultured porcine retinae. Retinal explants from the visual streak of porcine donor eyes are exposed to either siNeg or siCFH hTERT-RPE1 cells for 72 hours and medium was supplemented with purified FH or C3. Explants cryosections were stained with DAPI for nuclei and retinal architecture parameters were quantified: **A, D** retinal thickness, **B-E** ONL thickness and **C,F** number of cells in ONL. Abbreviations: GCL – Ganglion cell layer, INL – Inner nuclear layer, ONL – Outer nuclear layer, RPE – Retinal pigmented epithelium cells, siNeg – silencing negative control, si*CFH* – silencing *CFH*. The controls were identical for experiments in A,B,C and D,E,F. Mean ± SEM is shown. N= 3 biological replicates. *p ≤ 0.05; **p ≤0.005.

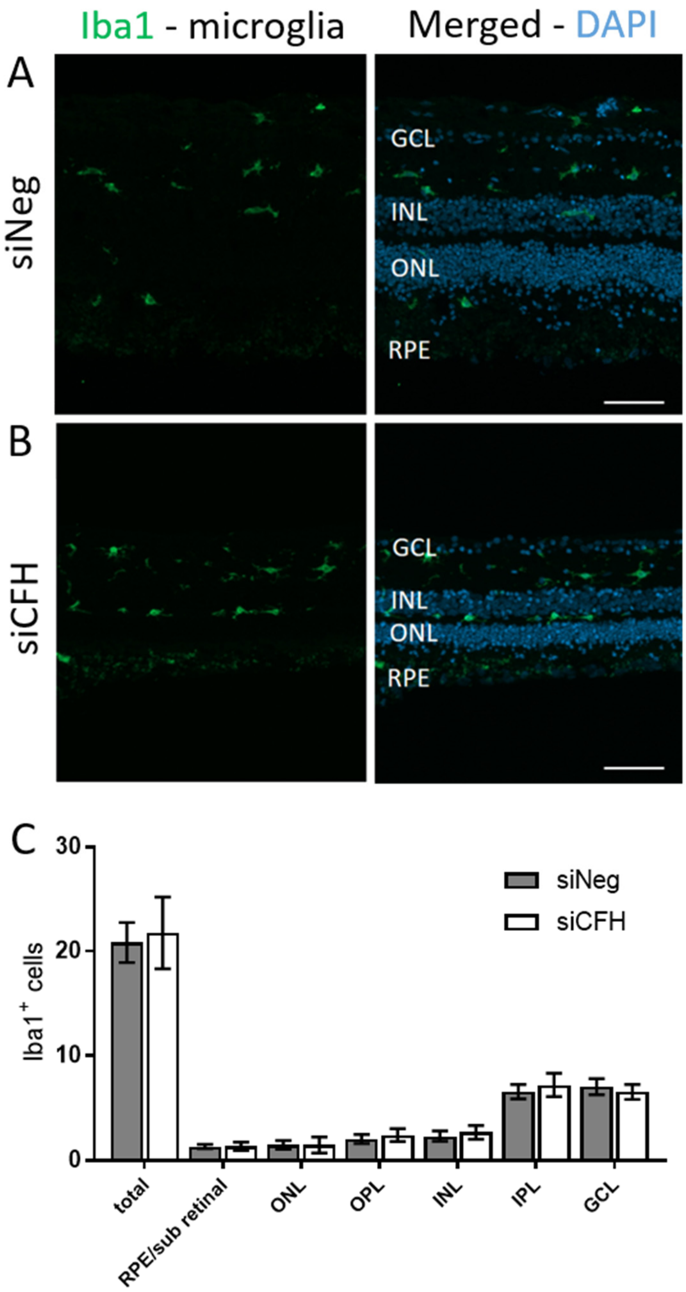

Figure S5: FH loss in RPE cells does not affect inflammatory cells on the co-cultured porcine retinae. Retinal explants from the visual streak of porcine donor eyes are exposed to either siNeg (A) or siCFH (B) hTERT-RPE1 cells for 72 hours. **A-B** Explants cryosections were stained for microglia marker Iba1 (green) and counterstaining was performed with DAPI for nuclei.. **C** Quantification of Iba1 positive cells extrapolated from A-B. Abbreviations: GCL – Ganglion cell layer, INL – Inner nuclear layer, ONL – Outer nuclear layer, RPE – Retinal pigmented epithelium cells, siNeg – silencing negative control, si*CFH* – silencing *CFH*. Mean ± SEM is shown. N= 6 biological replicates.

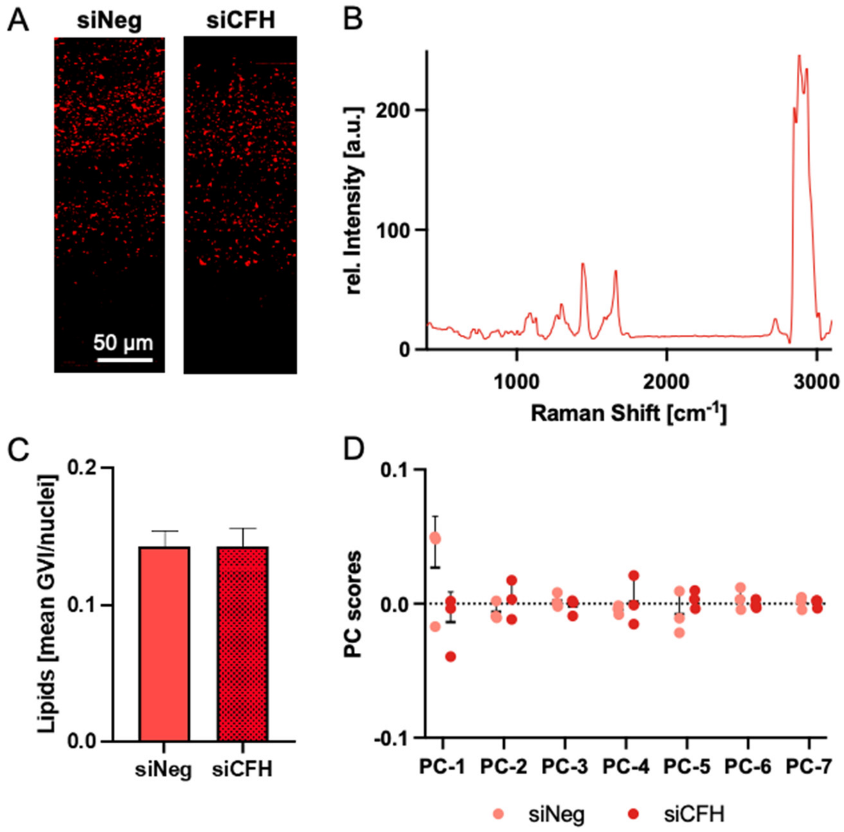

Figure S6: siCFH RPEs do not have an impact on the lipid expression and composition in the inner retina. **A-C** TCA identified a second lipid component localized in the inner retina (A) with a spectral signature assigned to phospholipids (B). Quantitative analysis by mean GVI/cell (C) did not show differences among both groups. **D** PCA of extracted lipid spectra expressed no significant difference in the lipid composition of the siNeg and siCFH group for any of the analyzed PCs. Data represent mean ± SD, n=3, scale bar – 50 µm

Raman Shift [cm^{-1}]	Molecular assignment	Literature
720	C-N (lipids)	34
750	pyrrole ring breathing	53
792	DNA	56, 57
1098	Phosphate backbone (DNA)	56,57
1130	C-C stretching	53
1270	C-H deformation	34
1450	CH_2 scissoring / CH_3 bending (lipids)	34
1580	NH_2 (DNA bases)	55
1589	C=C, C=N	53
1655	C=O stretching (lipids)	34
2840	CH_2 (lipids)	34
2850	CH_2 (lipids)	34
2940	CH_3 (lipids)	34

Table S1: Overview of Raman peaks and their molecular assignments.
